# Protease-activated receptor-1 (PAR-1): a promising molecular target for cancer

**DOI:** 10.18632/oncotarget.21015

**Published:** 2017-09-18

**Authors:** Xuan Liu, Jiahui Yu, Shangjin Song, Xiaoqiang Yue, Qi Li

**Affiliations:** ^1^ Department of Medical Oncology and Cancer Institute, Shuguang Hospital, Shanghai University of Traditional Chinese Medicine, Shanghai 201203, China; ^2^ Department of Traditional Chinese Medicine, Changzheng Hospital, Second Military Medical University, Shanghai 200003, China

**Keywords:** PAR-1, cancer, carcinogenesis, invasion, metastasis

## Abstract

PAR-1 is expressed not only in epithelium, neurons, astrocytes, immune cells, but also in cancer-associated fibroblasts, ECs (epithelial cells), myocytes of blood vessels, mast cells, and macrophages in tumor microenvironment, whereas PAR-1 stimulates macrophages to synthesize and secrete thrombin as well as other growth factors, resulting in enhanced cell proliferation, tumor growth and metastasis. Therefore, considerable effort has been devoted to the development of inhibitors targeting PAR-1. Here, we provide a comprehensive review of PAR-1’s role in cancer invasiveness and dissemination, as well as potential therapeutic strategies targeting PAR-1 signaling.

## INTRODUCTION

PAR-1 was the first member of the PARs (protease-activated receptors) family, which was found simultaneously by both two independent laboratories in 1991, during the process of identifying GPCR (G protein-coupled receptors) that mediate thrombin signal pathway in both human and hamster cells [1–3]. Thrombin-activated PAR-1 is expressed not only in all types of blood cells, but also in epithelium, neurons, astrocytes, and immune cells [3, 4, 5–7]. Furthermore, PAR-1 expression is also expressed in cancer-related fibroblasts, ECs (Epithelial Cells), blood vessels myocytes, mast cells, and macrophages in tumor microenvironment [8, 9]. In macrophages, PAR-1 elevates levels of numerous growth factors including thrombin [9]. More studies had since focused on the role of PAR-1 in biological function of tumor cells, as well as PAR-1 agonists and inhibitors [10–12]. PAR-1 as a target drug has become a hot spot in recent years, of which vorapaxar and atopaxar have entered the phase 3 clinical trial and phase 2 clinical trial, the clinical efficacy evaluation has become the last two years of research hotspots, which is expected to provide new clinical treatment ideas [13–23]. Hence, we review the role of PAR-1 in tumor development, invasion and metastasis, and discuss the potential therapeutic strategies for targeting PAR-1 signaling.

### Biological function of PAR-1

**Figure 1:**
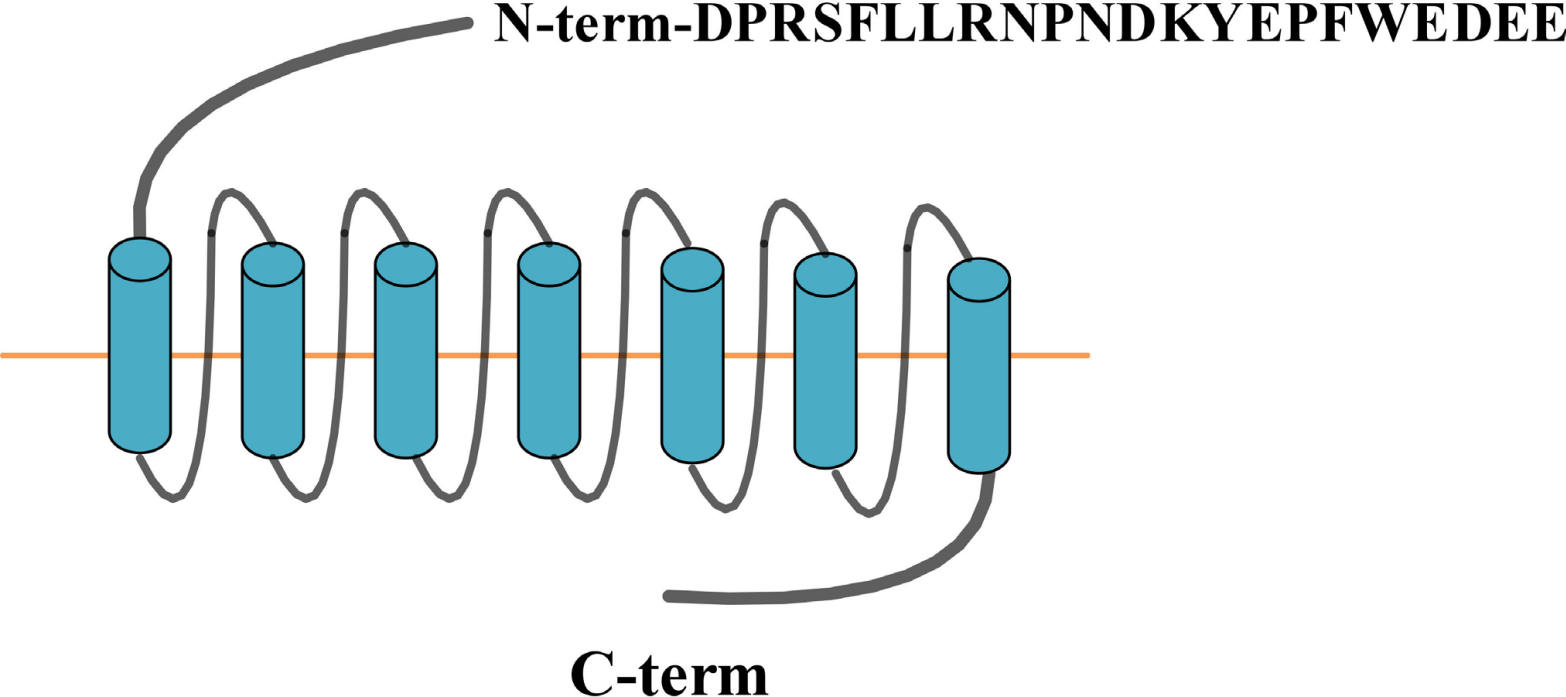
PAR-1 structure

PAR-1 is a G protein-coupled receptor consisting of 415 amino acids, five functional domains: extracellular N-terminal, extracellular loop, 7 hydrophobic transmembrane domain, intracellular loop and intracellular C-terminal (Figure 1). PAR-1 is irreversibly activated by thrombin, tissue factor (TF), endothelial protein C receptor (EPCR), MMPs, and so on. More and more evidence has showed that PAR-1 not only participates in normal biological functions, but also in tumorigenesis.

#### Activation

The binding of thrombin, principal ligand of PAR-1, to the N-terminus LDPR^41-^S^42^ sequence of the receptor cleaves the R^41-^S^42^ peptide bond [24]. The new unmasked sequence produced in this manner is used as a tethering ligand, which in turn binds intramolecular to the residue 42SFLLRN47 in the conserved region of the receptor second loop to induce transmembrane signaling. Matrix metalloprotease-1 (MMP-1) cleaves PAR-1 at a novel site (D^39-^P^40^) resulting in clonal ligands of two amino acids longer (PR-SFLLRN) than the one produced by thrombin, which activate the G12/13, Rho-GTP and MARK signaling to alter platelet shape and motility [3, 25]. EPCR interacts with the N-terminus of activated protein (APC) which induces protease cleavage of PAR-1 [26]. PAR-2 induced gene regulation by TF / FVIIa in glioblastoma cell line is mediated by thrombin-mediated activation of PAR-1 [27]. Of note, PAR-1’s activation is irreversible (Table 1).

**Table 1:** PAR-1 activators

PAR-1	Activators	The activation point
	Thrombin [3–7, 13]	R^41-^S^42^, S^42-^FLLRN^47^
	MMP-1,MMP-2,MMp-9,MMP-13 [3, 25]	D^39-^P^40^
	APC [26], Plasmin [26], Factor Xa [26], Granzyme A [26], Gingipains-R [26],	Cleave the N-terminus, with the EPCR as a cofactor
	TF-FVIIa [27]	Gene elcited by TF-FVIIa through PAR-2

#### Regulation

Two main mechanisms that account for activation (cleavage) of PAR-1 are receptor trafficking and desensitization [8]. PAR-1 transports from the cell membrane to the endosome, followed by degradation in lysosomes [28, 29]. PAR-1 internalization requires ubiquitination and is associated with the clathrin / AP2 (adapter protein 2) dimer and dynamin [30]. The transport of PAR-1 to lysosomes was facilitated by protein sorting nexin-1 (SNX-1) [31]. G protein-coupled receptor kinase (GPCRKs, GRKs) directed PAR-1 phosphorylation and protein interaction is fast, within a few seconds, ensued by G-protein dissociation and PAR-1 desensitization.

**Figure 2:**
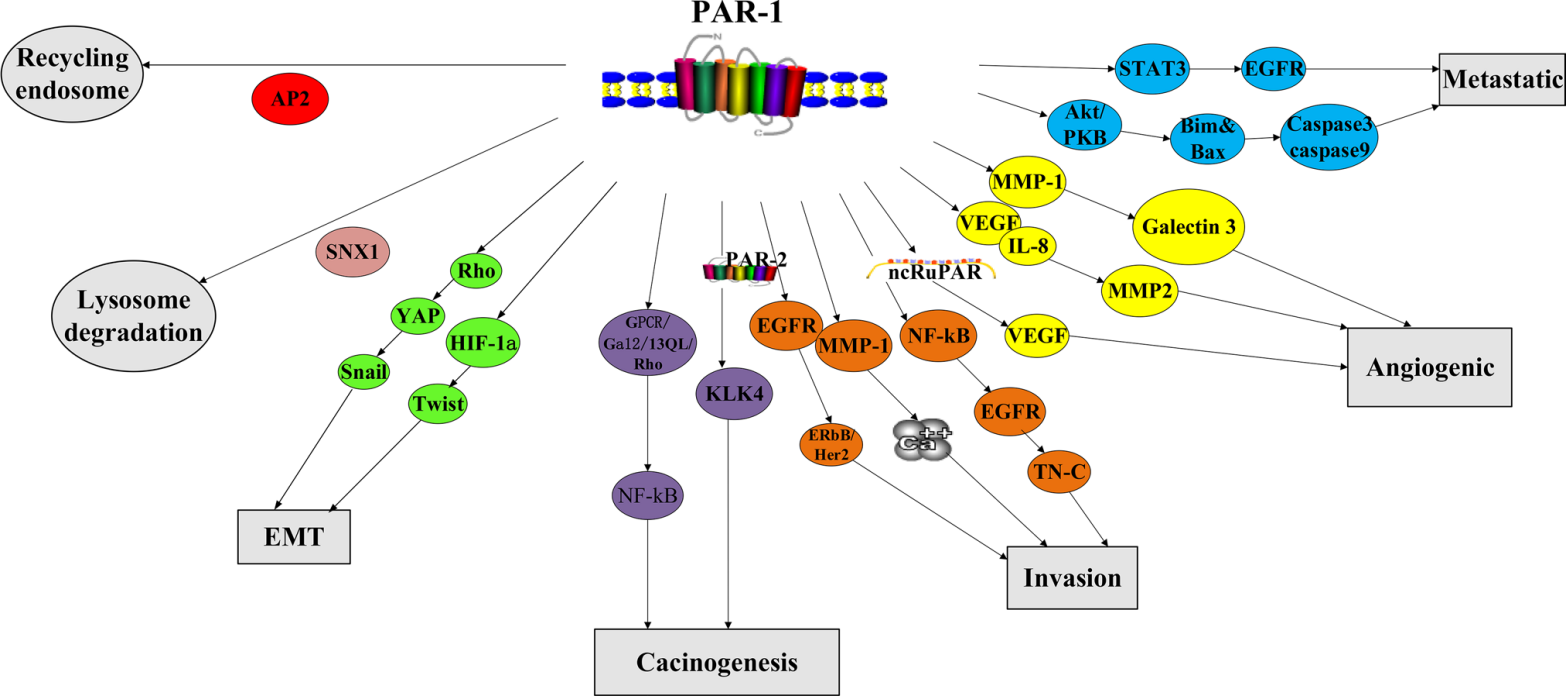
Biological function of PAR-1

In contrast to the tight and rapid control of PAR-1 activation in normal tissues, PAR-1 is constitutively activated in cancer cell (Figure 2). Thrombin activates signaling pathways in tumor cells by interacting with PAR-1 [33–35]. Most of the cellular responses are activated by the persistent stimulation of the second messenger ERK^1/2^ [36–37]. In a rat model of benign tumor, PAR-1 mediated silencing of pro-apoptotic genes led to tumor growth and invasion [38]. Repression of PAR-1 activity inhibited *in vivo* tumor growth, demonstrating PAR-1’s anti-apoptotic effects [36]. Consequently leading to consistent activation of second messenger signaling [36–37], PAR-1 cooperates with growth factor receptor (EGFR) and ErbB / Her2 or MMP-1 derived from fibroblasts to mediate Ca^2+^ pathway in cancer [39–40]. PAR-1 and MMP-1 alone can also up-regulate Galectin-3 [41]. PAR-1 signaling also interacts with the Hippo-YAP pathway to promote tumorigenesis [42].

PAR-1 is also involved in cancer cell invasion and metastasis (Figure 2). Multiple tumor cell lines show that PAR-1 overexpression is closely related to invasive phenotype and distant metastasis [33–34, 36, 37, 43–48]. PAR-1 enhances cancer cell invasiveness via increasing adhesion to extracellular matrix. After thrombin/PAR-1 stimulation, several cancer cell lines demonstrated increased platelets adhesion as well as to aorta and capillaries [32–34, 45, 49–50]. Prothrombin-induced HIF-1α increases mRNA expression of torsion, whose protein level is also mediated by activated PAR-1: all these can enhance EMT and increase tumor metastasis [42]. The interaction of cancer cells with integrin v5 and cytoskeleton promotes lung cancer and melanoma cell migration, invasion and metastasis [32, 50–51]. On the other hand, the use of anti-α_v_b_5_ antibodies specifically attenuated PAR-1-imediated invasion[50]. PAR-1 signaling induced expression of integrin IIb3 and P-selectin promoted melanoma cell-EC/platelet interaction, thereby increasing the metastatic potential of cancer cells [33–34, 45, 52–53]. Overexpression of NF-κB, EGFR can activate PAR-1 signaling, which consequently promotes tumor cell growth and invasion [54]. In contrast to normal tissue, STAT3-dependent transactivation of EGFR and PAR-1 in endothelial cells of clear cell renal cell carcinoma was significantly increased [55]. PAR-1 stimulated Akt / PKB signaling pathway, resulting in decreased Bim and Bax expression, and lower caspase-3 and caspase-9 cleavage levels, which induced less apoptosis [56].

PAR-1 plays an important role in angiogenesis (Figure 2). PAR-1 small interfering RNA (siRNA) lowered expression levels of IL-8, MMP-2 and VEGF, causing less vascular density [11]. PAR-1 expression is also directly associated with increased VEGF levels, stimulating angiogenesis [57]. PAR-1-induced effects depend on agonist concentration, allowing low concentrations of thrombin to stimulate the proliferation and growth of tumor cells, whereas high thrombin levels inducing apoptosis [58]. Down-regulation of long non-coding RNA-ncRuPAR resulted in tumor inhibition via modulating PAR-1 and VEGF [59]. Mouse development studies have confirmed the PAR-1-angiogenesis association since half of the mice that deprived PAR-1 perished due to poor blood development [60–62].

In summary, these aforementioned findings demonstrated that PAR-1-dependent promotion of tumor growth and metastasis is mediated by its regulation of adhesion and pro-antigenic factors, suggesting PAR-1 as a potential cancer therapeutic target.

### PAR-1 in cancers

**Figure 3:**
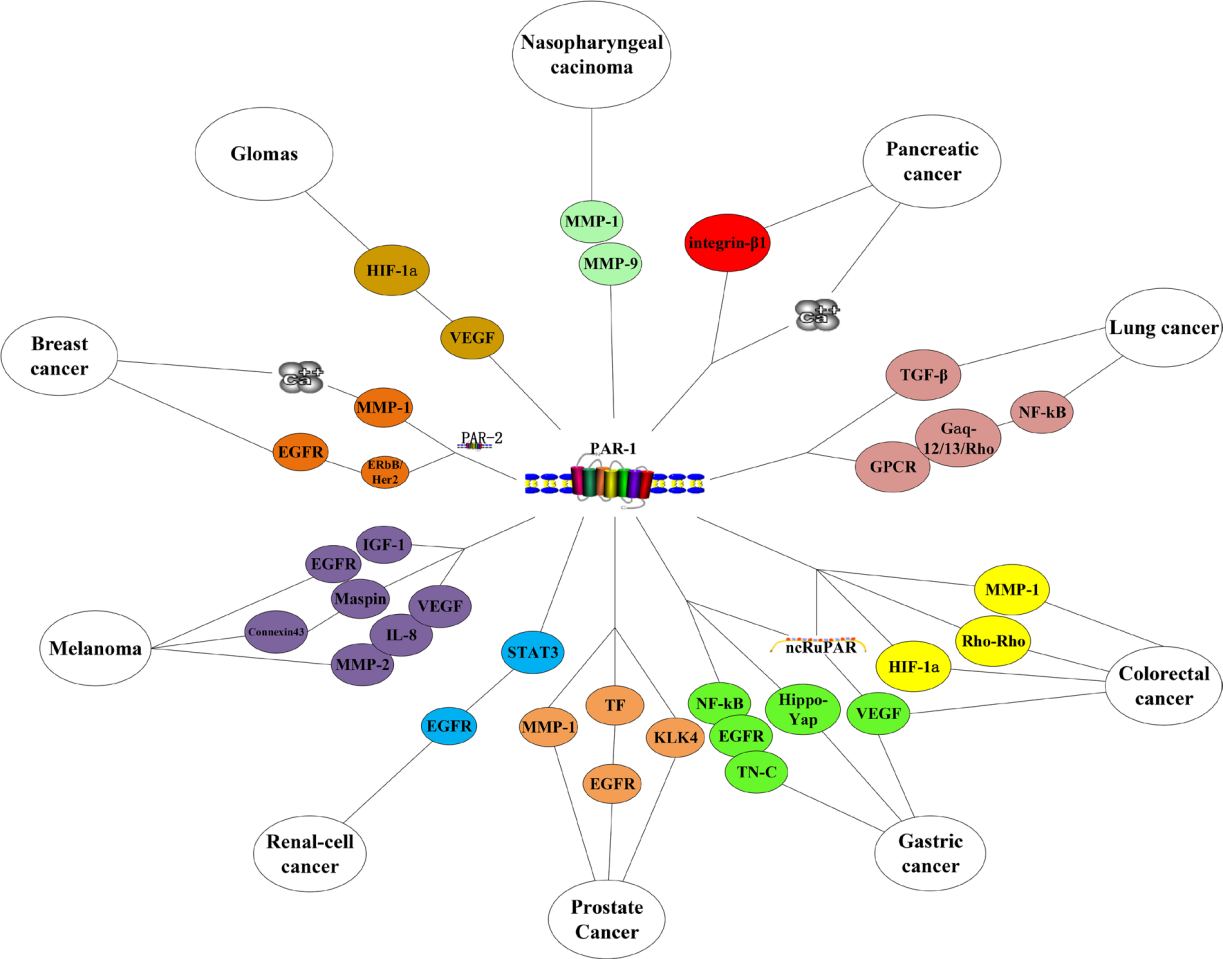
PAR-1 in cancers

Many a study has elucidated PAR-1 regulates several pro-tumorigenic signaling pathways in cancer. PAR-1 overexpression has been found in breast, melanoma, renal, gastric, colon, lung, pancreatic, esophageal, prostate, liver, ovarian, endometrial, head and neck cancers [27, 43, 46–47, 63–69] (Suppplementary Table 1, Figure 3).

#### Breast cancer

While not secreted in normal breast epithelium, benign dysplasia or adenoma, PAR-1 over-expresses *in situ* carcinoma and secreted in invasive breast cancer cell lines [38, 70–71]. PAR-1 signaling is activated by TF, MMPs and thrombin, mediates tumor progression, PAR-1 and PAR-2 cooperate functionally in breast cancer [8, 72]. Tumor growth and invasion in breast cancer gland xenograft models require thrombin-induced interplay between ErbB and EGFR, or by MMP-1-induced fibroblasts derived Ca^2+^ signaling [8]. Sustained activation of ErbB/Her2 and EGFR via thrombin-cleaved PAR-1 signaling was identified in invasive breast cancer but not in normal mammary epithelial cells [8, 36].

#### Melanoma

PAR-1 is over-expressed in metastatic melanoma cell lines and metastatic melanomas, but not in primary nevus and normal skin [11, 55]. In addition, melanoma cells isolated from patients’ metastatic lesions had increased PAR-1 mRNA and protein expression compared to those of non-metastatic disease [73]. Studies also revealed activated PAR-1 signal pathway in precursor phenotype of melanoma cells [11, 32, 40]. Studies on melanoma cell lines showed that PAR-1 signaling mobilized adhesion, invasion, anti-apoptotic and angiogenic factors to promote the invasion and metastasis of melanoma [11, 32, 40]. The migration capability of melanoma cells is enabled by thrombin- or MMP-1-mediated PAR-1 activation [40, 70, 74–75]. MMP-1 is shown to enhance type I collagen levels through skin to promote melanoma invasion, whereas PAR-1 activation leads to an increase in growth factor activation of EGFR and IGF-1 [40, 55]. In addition, PAR-1 induces metastatic melanoma by modulating tumor suppressor Maspin and the connexin 43 [76]. PAR-1 silencing and inhibiting thrombin decrease dissemination of metastatic melanoma cells [11–12, 77]. PAR-1 siRNA mediated inhibition decreased MMP-2, IL-8 and VEGF, expression levels, subsequently vascular density [78]. Accordingly, studies have shown that by inhibiting PAR-1 function, melanoma cells lost motility, became non-metastatic and less invasive.

#### Renal-cell cancer

It was reported that PAR-1 was associated with distant metastasis and survival in renal cell carcinoma (RCC). AA genotype of PAR-1 gene variant IVSn-14A> T was associated with an increased risk of RCC metastasis and a poorer prognosis [79]. In contrast to normal tissues, STAT3-dependent EGFR and PAR-1 activation in endothelial cells OF clear cell renal cell carcinoma was significantly increased [55].

#### Gastric and colorectal cancers

Thrombin-activated PAR-1 induces EMT (epithelial-mesenchymal transition, EMT) in gastric cancer cell lines [80]. Overexpression of NF-κB, EGFR, and TN-C also activated PAR-1 expression, which in turn promoted gastric cancer cell growth and invasion [54]. PAR-1 signaling is involved in multidrug resistance and tumorigenesis by interacting with Hippo-YAP pathway in gastric cancer stem cell-like cells [43]. EPCR activates ERK^1/2^ through PAR-1 to enhance proliferation and migration of MGC803 gastric cancer cells [37]. PAR-1 and MMP-1 up-regulate Galectin-3 in metastatic gastric cancer [41]. LncRNA-ncRuPAR regulates PAR-1 and VEGF in GC patients [59]. ALEX1 inhibits gastric cancer metastasis through dampening PAR-1/Rho GTPase signaling pathway [81]. PAR-1 expression levels are higher in metastatic gastric cancer and have prognostic value [82].

PAR-1 is associated with prognostic factors for colorectal cancer [83]. PAR-1 could promote colorectal cancer growth, local invasion and metastasis [84]. Downregulation of lncRNA-ncRuPAR contributes to tumor inhibition through PAR-1 and VEGF in colorectal cancer patients [85]. PAR-1 induced platelet activation is critical in EMT and migration of colon cancer cells [86]. Thrombin-mediated HIF-1α increases twist mRNA and protein levels, which is induced by PAR-1 activation and regulation of the HIF-1α translation, thereby regulating EMT and increasing metastasis [42]. KLK4 induces activated PAR-1 signaling in colon tumorigenesis [87]. Tumor-endothelial cross-talk via an intravascular MMP-1/PAR-1 axis exists in microvascular and macrovascular endothelium [88]. PAR-1 signaling enhances cancer cell invasion via Rho-Rho kinase axis and tumor microenvironment [89]. Activated PAR-1 also promotes colon cancer cell proliferation EGFR transactivation [90].

#### Lung cancer

The serum levels of PAR-1 might have a diagnostic value in lung cancer patients [91]. PAR-1 in NSCLC (Non-small cell lung cancer) is mainly expressed in cells that constitute the tumor microenvironment, including vascular endothelial cells, macrophages and stromal fibroblasts [92]. According to a survey of 209 patients, PAR-1 polymorphism was associated with tumor stage and median OS (overall survival) of squamous cell lung cancer patients [93]. A study of 63 lung cancer patients showed that continuous activation of platelets and thus exhaustion was involved in cancer-associated venous thromboembolism (VTE) and cancer mortality, through activating PAR-1 [94]. PAR-1 siRNA significantly decreases lung adenocarcinoma cell growth and invasion [95]. PAR-1 expression was up-regulated by TGF-β and indispensible for A549 lung adenocarcinoma cells [96]. Gαq and Gα13, coupled with PAR-1 as well as constitutively active GαqQL and Gα12/13QL mutants to stimulate SCLC (small cell lung cancer) to connect autocrine bombesin (BBS). BBS-induced activation of GPCR/Gαq-12/13/Rho-mediated NF-κB signaling un-regulates the activity of NF-κB response element in the Shh gene promoter [97].

#### Pancreatic cancer

PAR-1 expression levels are positively associated with disease progression and OS in pancreatic cancer [98–100]. Thrombin-activated PAR-1 can significantly enhance the integrin β1-specific adhesion of pancreatic cancer cells to vitronectin [101]. Nuclear Ca^2+^ signaling generated by trypsin and thrombin-PAR-1 pathway promote proliferation in pancreatic stellate cells (PSC) [39].

#### Prostate cancer

It is reported that PAR-1 is overexpressed in prostate cancer, may contribute to the malignant progression of prostate cance [102–103]. Unregulated PAR-1 expression in peritumoral stroma of prostate cancer patient is associated with biochemical recurrence. MMP-1 and PAR-1 coexpression with the clinicopathological characteristics and prognosis of patients with prostate cancer [103]. Tissue kallikrein (TK) promotes keratinocyte migration through activation of PAR-1 and transactivation of EGFR [104]. Evidence also showed for a novel double-paracrine mechanism whereby cancer epithelium produces KLK4 to activate PAR-1 in the surrounding stroma, which in-turn releases cytokines (IL-6) that stimulate cancer cells to proliferate and increase production of KLKs [105].

#### Others

PAR-1 promotes tumor cell growth and invasion in nasopharyngeal carcinoma [106–107]. Thrombin-induced PAR-1 activation breaks down extracellular matrix and basement membrane to increase MMP-1/-9 levels, which is closely related to nasopharyngeal carcinoma metastasis [106]. PAR-1 enhances acute myeloid leukemia leukemia stem cell activity and aggravates disease progression [108–109]. The expression of PAR-1 in esophageal squamous cell carcinoma was increased [110], to promote glioma cell malignancy and glioblastoma neoangiogenesis [111]. Thrombin activates PAR-1 expression, thus enabling tumor cell seeding and metastasis, giving rise to increased tumor cell growth and angiogenesis in glioblastoma [112]. Per HIF-α/VEGF pathway, PAR-1 maintains self-renewal and tumorigenicity of tumor-initiating progenitor cells (TPC) in gliomas, whilst inhibition of PAR-1 signaling slows down tumor progression [113–114]. PAR-1 and PAR-4 activate common promigratory signaling pathways in Hep3B liver carcinoma cells including activation of the receptor tyrosine kinases Met and PDGFR, the formation of ROS and the inactivation of PTP1B. However, PAR1/4-triggered Met and PDGFR transactivation seem to be mediated independently from the ROS-PTP1B signaling module [115]. PAR-1 has also been shown to be associated with the pathogenesis of ovarian cancer, which may be associated with PO-14 - tumor expression of coagulation proteases of the APC pathway [116].

### Drugs targeting PAR-1 in clinical use

According to the experimental research mentioned above, PAR-1 inhibitors may have the effect of inhibiting tumor cell proliferation, reducing invasion and metastasis, and anti-tumor angiogenesis. The development of drugs targeting PAR-1 has caused widespread concern. Currently, vorapaxar (SCH530348) and atopaxar (E5555) are the two clinical formulations of PAR-1 inhibitors [13–23],which have undergone extensive clinical development.

Vorapaxar is the first PAR-1 inhibitor approved for clinical use. Regarding to vorapaxar, phase 3 clinical trial data has been available since 2012, and the parent drug company Merck has filed for submission of approval to the US FDA, as well as the European Medicines Agency (EMA) [13–14]. Its main indication is the reduction in thrombotic cardiovascular events in patients with previous myocardial infaction or symptomatic peripheral artery disease. Numerous clinical studies have demonstrated that it plays an effective role in peripheral arterial disease, pulmonary hypertension, acute coronary syndrome, and so on [15–18]. It is regarded as a new approach to antiplatelet therapy. Vorapaxar was recently approved in two key jurisdictions: the FDA approved the drug for the reduction of thrombotic cardiovascular events in patients with a history of MI or with PAD, and EMA approved it for the reduction of thrombotic cardiovascular events in those with a history of MI [19]. But vorapaxar in cancer clinical research is still very few. A recent study showd that vorapaxar could inhibit epithelial ovarian cancer (EOC) progression in ovarian cancer [20]. No other researches had been reported of PAR-1’s role in other cancers. The most common side effect of vorapaxar is bleeding, which needs careful assessment in treatment.

Atopaxar hydrobromide is the second inhibitor used in clinical. It shows potent inhibitory effects on human platelet aggregation. Phase 2 clinical evidence is available for atopaxar administered in combination with ASA and/or P2Y_12_ receptor antagonists. These trials reported an increased bleeding risk [21–22] While, another case of the evidence on atopaxar came from LANCELOT phase 2 trials, which had two target populations , ACS (acute coronary syndrome) and CAD (coronary artery disease) [21–23]. The goals of these two studies were to look at the safety and tolerability of atopaxar in patients with ACS [22–23] The results showed no increases in any CURE bleeding between the combined (50 mg, 100 mg, 200 mg) atopaxar group and the control group (0.6% versus 3.3%; *P* = 0.125); there was also no statistically significant difference in the rate of TIMI bleeding in the combined atopaxar group versus the control group (19.4% versus 16.4%; *P* = 0.61).As a result, further research is needed to confirm its side effect. Currently, there is no reports in the study of Atopaxar in cancer.

Thus, PAR-1 inhibitors in cancer clinical study is still lacking, to be further enriched and assessed. The potential importance of PAR-1 target in cancer therapy is of concern. Whether it can play a clinical role in the development of tumor invasion and metastasis, angiogenesis, is still our attention and important research direction.

## CONCLUSIONS

PAR-1 has far-reaching significance in the mechanism of cancer research, as the earliest and most in-depth molecular of the PARs family. As mentioned above, PAR-1 actively participates in steps of cancer cell proliferation, invasion and metastasis which involve complex mechanisms. Therefore, more PAR-1 centered studies are in dire need, not only for elucidation of its tumorigenic functions, but also for its future use as a promising molecular target for clinical treatment. Although PAR-1 antagonists are known to be potent antiplatelet agents that are also complementary to other antiplatelet therapies, its role in clinical cancer treatment is still a mystery. Once it is demonstrated that PAR-1 targeted drugs play a role in tumor development or invasion and metastasis, it may become a new target for tumor therapy. Drug research and development based on PAR-1 mechanism is still a new potential direction of clinical treatment.

## SUPPLEMENTARY MATERIALS TABLE





## References

[1] Rasmussen UB, Vouret-Craviari V, Jallat S, Schlesinger Y, Pagès G, Pavirani A, Lecocq JP, Pouysségur J, Van Obberghen-Schilling E (1991). cDNA cloning and expression of a hamster alpha-thrombin receptor coupled to Ca2+ mobilization. FEBS Lett.

[2] Vu TK, Hung DT, Wheaton VI, Coughlin SR (1991). Molecular cloning of a functional thrombin receptor reveals a novel proteolytic mechanism of receptor activation. Cell.

[3] Austin KM, Covic L, Kuliopulos A (2013). Matrix metalloproteases and PAR1 activation. Blood.

[4] Coughlin SR (2005). Protease-activated receptors in hemostasis, thrombosis and vascular biology. J Thromb Haemost.

[5] Nieman MT (2015). PARtitioning protease signaling. Blood.

[6] Lin H, Liu AP, Smith TH, Trejo J (2013). Cofactoring and dimerization of proteinase-activated receptors. Pharmacol Rev.

[7] Aisiku O, Peters CG, De Ceunynck K, Ghosh CC, Dilks JR, Fustolo-Gunnink SF, Huang M, Dockendorff C, Parikh SM, Flaumenhaft R (2015). Parmodulins inhibit thrombus formation without inducing endothelial injury caused by vorapaxar. Blood.

[8] Arora P, Ricks TK, Trejo J (2007). Protease-activated receptor signalling, endocytic sorting and dysregulation in cancer. J Cell Sci.

[9] White MJ, Gomer RH (2015). Trypsin, Tryptase, and Thrombin Polarize Macrophages towards a Pro-Fibrotic M2a Phenotype. PLoS One.

[10] Yang E, Boire A, Agarwal A, Nguyen N, O'Callaghan K, Tu P, Kuliopulos A, Covic L (2009). Blockade of PAR1 signaling with cell-penetrating pepducins inhibits Akt survival pathways in breast cancer cells and suppresses tumor survival and metastasis. Cancer Res.

[11] Villares GJ, Zigler M, Wang H, Melnikova VO, Wu H, Friedman R, Leslie MC, Vivas-Mejia PE, Lopez-Berestein G, Sood AK, Bar-Eli M (2008). Targeting melanoma growth and metastasis with systemic delivery of liposome-incorporated protease-activated receptor-1 small interfering RNA. Cancer Res.

[12] Tatour M, Shapira M, Axelman E, Ghanem S, Keren-Politansky A, Bonstein L, Brenner B, Nadir Y (2017). Thrombin is a selective inducer of heparanase release from platelets and granulocytes via protease-activated receptor-1. Thromb Haemost.

[13] Baker NC, Lipinski MJ, Lhermusier T, Waksman R (2014). Overview of the 2014 Food and Drug Administration Cardiovascular and Renal Drugs Advisory Committee meeting about vorapaxar. Circulation.

[14] Wang A (2015). Review of vorapaxar for the prevention of atherothrombotic events. Expert Opin Pharmacother.

[15] Klonaris C, Patelis N, Drebes A, Matheiken S, Liakakos T (2016). Antiplatelet Treatment in Peripheral Arterial Disease: The Role of Novel Antiplatelet Agents. Curr Pharm Des.

[16] Niespialowska-Steuden M, Collins P, Costopoulos C, Gorog DA (2014). NOAC in acute coronary syndrome and AF?. Cardiovasc Hematol Disord Drug Targets.

[17] Cunningham M, McIntosh K, Bushell T, Sloan G, Plevin R (2016). Proteinase-activated receptors (PARs) as targets for antiplatelet therapy. Biochem Soc Trans.

[18] Moschonas IC, Goudevenos JA, Tselepis AD (2015). Protease-activated receptor-1 antagonists in long-term antiplatelet therapy. Current state of evidence and future perspectives. Int J Cardiol.

[19] (2017). Smokers under 50 have eightfold increased risk of heart attack. Nurs Stand.

[20] Chanakira A, Westmark PR, Ong IM, Sheehan JP (2017). Tissue factor-factor VIIa complex triggers protease activated receptor 2-dependent growth factor release and migration in ovarian cancer. Gynecol Oncol.

[21] Wiviott SD, Flather MD, O'Donoghue ML, Goto S, Fitzgerald DJ, Cura F, Aylward P, Guetta V, Dudek D, Contant CF, Angiolillo DJ, Bhatt DL, LANCELOT-CAD Investigators (2011). Randomized trial of atopaxar in the treatment of patients with coronary artery disease: the lessons from antagonizing the cellular effect of Thrombin-Coronary Artery Disease Trial. Circulation.

[22] Goto S, Ogawa H, Takeuchi M, Flather MD, Bhatt DL, Investigators JL (2010). Double-blind, placebo-controlled Phase II studies of the protease-activated receptor 1 antagonist E5555 (atopaxar) in Japanese patients with acute coronary syndrome or high-risk coronary artery disease. Eur Heart J.

[23] O'Donoghue ML, Bhatt DL, Wiviott SD, Goodman SG, Fitzgerald DJ, Angiolillo DJ, Goto S, Montalescot G, Zeymer U, Aylward PE, Guetta V, Dudek D, Ziecina R (2011). Safety and tolerability of atopaxar in the treatment of patients with acute coronary syndromes: the lessons from antagonizing the cellular effects of Thrombin-Acute Coronary Syndromes Trial. Circulation.

[24] Yuan SM, Nie WC, He F, Jia ZW, Gao XD (2016). Kin2, the Budding Yeast Ortholog of Animal MARK/PAR-1 Kinases, Localizes to the Sites of Polarized Growth and May Regulate Septin Organization and the Cell Wall. PLoS One.

[25] Trivedi V, Boire A, Tchernychev B, Kaneider NC, Leger AJ, O'Callaghan K, Covic L, Kuliopulos A (2009). Platelet matrix metalloprotease-1 mediates thrombogenesis by activating PAR1 at a cryptic ligand site. Cell.

[26] Malaquin N, Vercamer C, Bouali F, Martien S, Deruy E, Wernert N, Chwastyniak M, Pinet F, Abbadie C, Pourtier A (2013). Senescent fibroblasts enhance early skin carcinogenic events via a paracrine MMP-PAR-1 axis. PLoS One.

[27] Albrektsen T, Sorensen BB, Hjorto GM, Fleckner J, Rao LV, Petersen LC (2007). Transcriptional program induced by factor VIIa-tissue factor, PAR1 and PAR2 in MDA-MB-231 cells. J Thromb Haemost.

[28] Hudak R, Vincze J, Csernoch L, Beke Debreceni I, Olah T, Erdodi F, Clemetson KJ, Kappelmayer J (2017). The Phosphatase Inhibitor Calyculin-A Impairs Clot Retraction, Platelet Activation, and Thrombin Generation. Biomed Res Int.

[29] Grimsey N, Lin H, Trejo J (2014). Endosomal signaling by protease-activated receptors. Methods Enzymol.

[30] Soh UJ, Dores MR, Chen B, Trejo J (2010). Signal transduction by protease-activated receptors. Br J Pharmacol.

[31] Gullapalli A, Wolfe BL, Griffin CT, Magnuson T, Trejo J (2006). An essential role for SNX1 in lysosomal sorting of protease-activated receptor-1: evidence for retromer-, Hrs-, and Tsg101-independent functions of sorting nexins. Mol Biol Cell.

[32] Nierodzik ML, Karpatkin S (2006). Thrombin induces tumor growth, metastasis, and angiogenesis: Evidence for a thrombin-regulated dormant tumor phenotype. Cancer Cell.

[33] Saleiban A, Faxalv L, Claesson K, Jonsson JI, Osman A (2014). miR-20b regulates expression of proteinase-activated receptor-1 (PAR-1) thrombin receptor in melanoma cells. Pigment Cell Melanoma Res.

[34] Zigler M, Kamiya T, Brantley EC, Villares GJ, Bar-Eli M (2011). PAR-1 and thrombin: the ties that bind the microenvironment to melanoma metastasis. Cancer Res.

[35] Villares GJ, Zigler M, Bar-Eli M (2011). The emerging role of the thrombin receptor (PAR-1) in melanoma metastasis--a possible therapeutic target. Oncotarget.

[36] Tatour M, Shapira M, Axelman E, Ghanem S, Keren-Politansky A, Bonstein L, Brenner B, Nadir Y (2017). Thrombin is a selective inducer of heparanase release from platelets and granulocytes via protease-activated receptor-1. Thromb Haemost.

[37] Wang Q, Liu Q, Wang T, Yang H, Han Z, Zhang P (2015). Endothelial cell protein C receptor promotes MGC803 gastric cancer cells proliferation and migration by activating ERK1/2. Med Oncol.

[38] Even-Ram S, Uziely B, Cohen P, Grisaru-Granovsky S, Maoz M, Ginzburg Y, Reich R, Vlodavsky I, Bar-Shavit R (1998). Thrombin receptor overexpression in malignant and physiological invasion processes. Nat Med.

[39] Won JH, Zhang Y, Ji B, Logsdon CD, Yule DI (2011). Phenotypic changes in mouse pancreatic stellate cell Ca2+ signaling events following activation in culture and in a disease model of pancreatitis. Mol Biol Cell.

[40] Zigler M, Kamiya T, Brantley EC, Villares GJ, Bar-Eli M (2011). PAR-1 and thrombin: the ties that bind the microenvironment to melanoma metastasis. Cancer Res.

[41] Kim SJ, Shin JY, Lee KD, Bae YK, Choi IJ, Park SH, Chun KH (2011). Galectin-3 facilitates cell motility in gastric cancer by up-regulating protease-activated receptor-1 (PAR-1) and matrix metalloproteinase-1 (MMP-1). PLoS One.

[42] Chang LH, Chen CH, Huang DY, Pai HC, Pan SL, Teng CM (2011). Thrombin induces expression of twist and cell motility via the hypoxia-inducible factor-1alpha translational pathway in colorectal cancer cells. J Cell Physiol.

[43] Sedda S, Marafini I, Caruso R, Pallone F, Monteleone G (2014). Proteinase activated-receptors-associated signaling in the control of gastric cancer. World J Gastroenterol.

[44] Fujimoto D, Hirono Y, Goi T, Katayama K, Yamaguchi A (2008). Prognostic value of protease-activated receptor-1 (PAR-1) and matrix metalloproteinase-1 (MMP-1) in gastric cancer. Anticancer Res.

[45] Moon JY, Franchi F, Rollini F, Angiolillo DJ Role for Thrombin Receptor Antagonism With Vorapaxar in Secondary Prevention of Atherothrombotic Events: From Bench to Bedside. J Cardiovasc Pharmacol Ther.

[46] Whetstone WD, Walker B, Trivedi A, Lee S, Noble-Haeusslein LJ, Hsu JC (2017). Protease-Activated Receptor-1 Supports Locomotor Recovery by Biased Agonist Activated Protein C after Contusive Spinal Cord Injury. PLoS One.

[47] Otsuki T, Fujimoto D, Hirono Y, Goi T, Yamaguchi A (2014). Thrombin conducts epithelialmesenchymal transition via proteaseactivated receptor1 in human gastric cancer. Int J Oncol.

[48] Borensztajn KS, Bijlsma MF, Groot AP, Bruggemann LW, Versteeg HH, Reitsma PH, Peppelenbosch MP, Spek CA (2007). Coagulation factor Xa drives tumor cells into apoptosis through BH3-only protein Bim up-regulation. Exp Cell Res.

[49] Mahajan VB, Pai KS, Lau A, Cunningham DD (2000). Creatine kinase, an ATP-generating enzyme, is required for thrombin receptor signaling to the cytoskeleton. Proc Natl Acad Sci U S A.

[50] Even-Ram SC, Maoz M, Pokroy E, Reich R, Katz BZ, Gutwein P, Altevogt P, Bar-Shavit R (2001). Tumor cell invasion is promoted by activation of protease activated receptor-1 in cooperation with the alpha vbeta 5 integrin. J Biol Chem.

[51] Bai SY, Xu N, Chen C, Song YL, Hu J, Bai CX (2015). Integrin alphavbeta5 as a biomarker for the assessment of non-small cell lung cancer metastasis and overall survival. Clin Respir J.

[52] Trikha M, Timar J, Zacharek A, Nemeth JA, Cai Y, Dome B, Somlai B, Raso E, Ladanyi A, Honn KV (2002). Role for beta3 integrins in human melanoma growth and survival. Int J Cancer.

[53] Raso E, Tovari J, Toth K, Paku S, Trikha M, Honn KV, Timar J (2001). Ectopic alphaIIbbeta3 integrin signaling involves 12-lipoxygenase- and PKC-mediated serine phosphorylation events in melanoma cells. Thromb Haemost.

[54] Amador MA, Cavalcante GC, Santos NP, Gusmao L, Guerreiro JF, Ribeiro-dos-Santos A, Santos S (2016). Distribution of allelic and genotypic frequencies of IL1A, IL4, NFKB1 and PAR1 variants in Native American, African, European and Brazilian populations. BMC Res Notes.

[55] Massi D, Naldini A, Ardinghi C, Carraro F, Franchi A, Paglierani M, Tarantini F, Ketabchi S, Cirino G, Hollenberg MD, Geppetti P, Santucci M (2005). Expression of protease-activated receptors 1 and 2 in melanocytic nevi and malignant melanoma. Hum Pathol.

[56] Maeda S, Nakajima K, Tohyama Y, Kohsaka S (2009). Characteristic response of astrocytes to plasminogen/plasmin to upregulate transforming growth factor beta 3 (TGFbeta3) production/secretion through proteinase-activated receptor-1 (PAR-1) and the downstream phosphatidylinositol 3-kinase (PI3K)-Akt/PKB signaling cascade. Brain Res.

[57] Yin YJ, Salah Z, Maoz M, Even Ram SC, Ochayon S, Neufeld G, Katzav S, Bar-Shavit R (2003). Oncogenic transformation induces tumor angiogenesis: a role for PAR1 activation. FASEB J.

[58] Zain J, Huang YQ, Feng X, Nierodzik ML, Li JJ, Karpatkin S (2000). Concentration-dependent dual effect of thrombin on impaired growth/apoptosis or mitogenesis in tumor cells. Blood.

[59] Liu L, Yan B, Yang Z, Zhang X, Gu Q, Yue X (2014). ncRuPAR inhibits gastric cancer progression by down-regulating protease-activated receptor-1. Tumour Biol.

[60] Xie Q, Bao X, Chen ZH, Xu Y, Keep RF, Muraszko KM, Xi G, Hua Y (2016). Role of Protease-Activated Receptor-1 in Glioma Growth. Acta Neurochir Suppl.

[61] Huang Z, Miao X, Luan Y, Zhu L, Kong F, Lu Q, Pernow J, Nilsson G, Li N (2015). PAR1-stimulated platelet releasate promotes angiogenic activities of endothelial progenitor cells more potently than PAR4-stimulated platelet releasate. J Thromb Haemost.

[62] Reinhardt C, Bergentall M, Greiner TU, Schaffner F, Ostergren-Lunden G, Petersen LC, Ruf W, Backhed F (2012). Tissue factor and PAR1 promote microbiota-induced intestinal vascular remodelling. Nature.

[63] Morris DR, Ding Y, Ricks TK, Gullapalli A, Wolfe BL, Trejo J (2006). Protease-activated receptor-2 is essential for factor VIIa and Xa-induced signaling, migration, and invasion of breast cancer cells. Cancer Res.

[64] Fujimoto D, Hirono Y, Goi T, Katayama K, Matsukawa S, Yamaguchi A (2010). The activation of Proteinase-Activated Receptor-1 (PAR1) mediates gastric cancer cell proliferation and invasion. BMC Cancer.

[65] Uzunoglu FG, Yavari N, Bohn BA, Nentwich MF, Reeh M, Pantel K, Perez D, Tsui TY, Bockhorn M, Mann O, Izbicki JR, Wikman H, Vashist YK (2013). C-X-C motif receptor 2, endostatin and proteinase-activated receptor 1 polymorphisms as prognostic factors in NSCLC. Lung Cancer.

[66] Uzunoglu FG, Kolbe J, Wikman H, Gungor C, Bohn BA, Nentwich MF, Reeh M, Konig AM, Bockhorn M, Kutup A, Mann O, Izbicki JR, Vashist YK (2013). VEGFR-2, CXCR-2 and PAR-1 germline polymorphisms as predictors of survival in pancreatic carcinoma. Ann Oncol.

[67] Li SM, Jiang P, Xiang Y, Wang WW, Zhu YC, Feng WY, Li SD, Yu GY (2014). Protease-activated receptor (PAR)1, PAR2 and PAR4 expressions in esophageal squamous cell carcinoma. Dongwuxue Yanjiu.

[68] Liao M, Tong P, Zhao J, Zhang Y, Li Z, Wang J, Feng X, Hu M, Pan Y (2012). Prognostic value of matrix metalloproteinase-1/ proteinase-activated receptor-1 signaling axis in hepatocellular carcinoma. Pathol Oncol Res.

[69] Yin YJ, Salah Z, Grisaru-Granovsky S, Cohen I, Even-Ram SC, Maoz M, Uziely B, Peretz T, Bar-Shavit R (2003). Human protease-activated receptor 1 expression in malignant epithelia: a role in invasiveness. Arterioscler Thromb Vasc Biol.

[70] Vianello F, Sambado L, Goss A, Fabris F, Prandoni P (2016). Dabigatran antagonizes growth, cell-cycle progression, migration, and endothelial tube formation induced by thrombin in breast and glioblastoma cell lines. Cancer Med.

[71] Jaber M, Maoz M, Kancharla A, Agranovich D, Peretz T, Grisaru-Granovsky S, Uziely B, Bar-Shavit R (2014). Protease-activated-receptor-2 affects protease-activated-receptor-1-driven breast cancer. Cell Mol Life Sci.

[72] Ohshiro K, Bui-Nguyen TM, Divijendra Natha RS, Schwartz AM, Levine P, Kumar R (2012). Thrombin stimulation of inflammatory breast cancer cells leads to aggressiveness via the EGFR-PAR1-Pak1 pathway. Int J Biol Markers.

[73] Silini A, Ghilardi C, Ardinghi C, Bernasconi S, Oliva P, Carraro F, Naldini A, Bani MR, Giavazzi R (2010). Protease-activated receptor-1 (PAR-1) promotes the motility of human melanomas and is associated to their metastatic phenotype. Clin Exp Metastasis.

[74] Blackburn JS, Liu I, Coon CI, Brinckerhoff CE (2009). A matrix metalloproteinase-1/protease activated receptor-1 signaling axis promotes melanoma invasion and metastasis. Oncogene.

[75] Villares GJ, Zigler M, Bar-Eli M (2011). The emerging role of the thrombin receptor (PAR-1) in melanoma metastasis--a possible therapeutic target. Oncotarget.

[76] Melnikova VO, Balasubramanian K, Villares GJ, Dobroff AS, Zigler M, Wang H, Petersson F, Price JE, Schroit A, Prieto VG, Hung MC, Bar-Eli M (2009). Crosstalk between protease-activated receptor 1 and platelet-activating factor receptor regulates melanoma cell adhesion molecule (MCAM/MUC18) expression and melanoma metastasis. J Biol Chem.

[77] Waitkus MS, Chandrasekharan UM, Willard B, Tee TL, Hsieh JK, Przybycin CG, Rini BI, Dicorleto PE (2014). Signal integration and gene induction by a functionally distinct STAT3 phosphoform. Mol Cell Biol.

[78] Villares GJ, Zigler M, Wang H, Melnikova VO, Wu H, Friedman R, Leslie MC, Vivas-Mejia PE, Lopez-Berestein G, Sood AK, Bar-Eli M (2008). Targeting melanoma growth and metastasis with systemic delivery of liposome-incorporated protease-activated receptor-1 small interfering RNA. Cancer Res.

[79] Otsuki T, Fujimoto D, Hirono Y, Goi T, Yamaguchi A (2014). Thrombin conducts epithelialmesenchymal transition via proteaseactivated receptor1 in human gastric cancer. Int J Oncol.

[80] Pang L, Li JF, Su L, Zang M, Fan Z, Yu B, Wu X, Li C, Yan M, Zhu ZG, Liu B (2017). ALEX1, a novel tumor suppressor gene, inhibits gastric cancer metastasis via the PAR-1/Rho GTPase signaling pathway. J Gastroenterol.

[81] Clouston HW, Davenport A, Gregson H, Shaker H, Duff S, Kirwan CC (2016). PO-51 - Expression of proteins of the tissue factor thrombin pathway is upregulated in the stroma and epithelium of colorectal cancer. Thromb Res.

[82] Tas F, Karabulut S, Tastekin D, Duranyildiz D (2016). Clinical significance of serum protease-activated receptor-1 levels in gastric cancer patients. Biomed Rep.

[83] Adams GN, Rosenfeldt L, Frederick M, Miller W, Waltz D, Kombrinck K, McElhinney KE, Flick MJ, Monia BP, Revenko AS, Palumbo JS (2015). Colon Cancer Growth and Dissemination Relies upon Thrombin, Stromal PAR-1, and Fibrinogen. Cancer Res.

[84] Yan B, Gu W, Yang Z, Gu Z, Yue X, Gu Q, Liu L (2014). Downregulation of a long noncoding RNA-ncRuPAR contributes to tumor inhibition in colorectal cancer. Tumour Biol.

[85] Jia Y, Zhang S, Miao L, Wang J, Jin Z, Gu B, Duan Z, Zhao Z, Ma S, Zhang W, Li Z (2015). Activation of platelet protease-activated receptor-1 induces epithelial-mesenchymal transition and chemotaxis of colon cancer cell line SW620. Oncol Rep.

[86] Gratio V, Beaufort N, Seiz L, Maier J, Virca GD, Debela M, Grebenchtchikov N, Magdolen V, Darmoul D (2010). Kallikrein-related peptidase 4: a new activator of the aberrantly expressed protease-activated receptor 1 in colon cancer cells. Am J Pathol.

[87] Goerge T, Barg A, Schnaeker EM, Poppelmann B, Shpacovitch V, Rattenholl A, Maaser C, Luger TA, Steinhoff M, Schneider SW (2006). Tumor-derived matrix metalloproteinase-1 targets endothelial proteinase-activated receptor 1 promoting endothelial cell activation. Cancer Res.

[88] Nguyen QD, De Wever O, Bruyneel E, Hendrix A, Xie WZ, Lombet A, Leibl M, Mareel M, Gieseler F, Bracke M, Gespach C (2005). Commutators of PAR-1 signaling in cancer cell invasion reveal an essential role of the Rho-Rho kinase axis and tumor microenvironment. Oncogene.

[89] Darmoul D, Gratio V, Devaud H, Peiretti F, Laburthe M (2004). Activation of proteinase-activated receptor 1 promotes human colon cancer cell proliferation through epidermal growth factor receptor transactivation. Mol Cancer Res.

[90] Erturk K, Tastekin D, Bilgin E, Tas F, Disci R, Duranyildiz D (2016). Clinical significance of serum protease activated receptor1 levels in patients with lung cancer. Eur Rev Med Pharmacol Sci.

[91] Lin C, Majoor CJ, Roelofs JJ, de Kruif MD, Horlings HM, Borensztajn K, Spek CA (2017). Potential importance of protease activated receptor (PAR)-1 expression in the tumor stroma of non-small-cell lung cancer. BMC Cancer.

[92] Uzunoglu FG, Yavari N, Bohn BA, Nentwich MF, Reeh M, Pantel K, Perez D, Tsui TY, Bockhorn M, Mann O, Izbicki JR, Wikman H, Vashist YK (2013). C-X-C motif receptor 2, endostatin and proteinase-activated receptor 1 polymorphisms as prognostic factors in NSCLC. Lung Cancer.

[93] Riedl J, Kaider A, Marosi C, Prager G, Eichelberger B, Koder S, Panzer S, Pabinger I, Ay C (2016). PO-63 - Exhausted platelets in cancer patients with high risk of venous thromboembolism and poor prognosis. Thromb Res.

[94] Wu Z, Zeng Y, Zhong M, Wang B (2014). Targeting A549 lung adenocarcinoma cell growth and invasion with proteaseactivated receptor1 siRNA. Mol Med Rep.

[95] Smoktunowicz N, Plate M, Stern AO, D'Antongiovanni V, Robinson E, Chudasama V, Caddick S, Scotton CJ, Jarai G, Chambers RC (2016). TGFbeta upregulates PAR-1 expression and signalling responses in A549 lung adenocarcinoma cells. Oncotarget.

[96] Castellone MD, Laukkanen MO, Teramoto H, Bellelli R, Ali G, Fontanini G, Santoro M, Gutkind JS (2015). Cross talk between the bombesin neuropeptide receptor and Sonic hedgehog pathways in small cell lung carcinoma. Oncogene.

[97] Queiroz KC, Shi K, Duitman J, Aberson HL, Wilmink JW, van Noesel CJ, Richel DJ, Spek CA (2014). Protease-activated receptor-1 drives pancreatic cancer progression and chemoresistance. Int J Cancer.

[98] Uzunoglu FG, Kolbe J, Wikman H, Gungor C, Bohn BA, Nentwich MF, Reeh M, Konig AM, Bockhorn M, Kutup A, Mann O, Izbicki JR, Vashist YK (2013). VEGFR-2, CXCR-2 and PAR-1 germline polymorphisms as predictors of survival in pancreatic carcinoma. Ann Oncol.

[99] Queiroz KC, Shi K, Duitman J, Aberson HL, Wilmink JW, van Noesel CJ, Richel DJ, Spek CA (2014). Protease-activated receptor-1 drives pancreatic cancer progression and chemoresistance. Int J Cancer.

[100] Kanemaru M, Maehara N, Iwamura T, Chijiiwa K (2012). Thrombin stimulates integrin beta1-dependent adhesion of human pancreatic cancer cells to vitronectin through protease-activated receptor (PAR)-1. Hepatogastroenterology.

[101] Wang H, Yi T, Zheng Y, He S (2007). Induction of monocyte chemoattractant protein-1 release from A549 cells by agonists of protease-activated receptor-1 and -2. Eur J Cell Biol.

[102] Zhang X, Wang W, True LD, Vessella RL, Takayama TK (2009). Protease-activated receptor-1 is upregulated in reactive stroma of primary prostate cancer and bone metastasis. Prostate.

[103] Wang J, Liu D, Zhou W, Wang M, Xia W, Tang Q (2014). Prognostic value of matrix metalloprotease-1/protease-activated receptor-1 axis in patients with prostate cancer. Med Oncol.

[104] Gao L, Smith RS, Chen LM, Chai KX, Chao L, Chao J (2010). Tissue kallikrein promotes prostate cancer cell migration and invasion via a protease-activated receptor-1-dependent signaling pathway. Biol Chem.

[105] Wang W, Mize GJ, Zhang X, Takayama TK (2010). Kallikrein-related peptidase-4 initiates tumor-stroma interactions in prostate cancer through protease-activated receptor-1. Int J Cancer.

[106] Yang R, Xu Y, Li P, Zhang X, Wang J, Gu D, Wang Y (2013). Combined upregulation of matrix metalloproteinase-1 and proteinase-activated receptor-1 predicts unfavorable prognosis in human nasopharyngeal carcinoma. Onco Targets Ther.

[107] Zhu Q, Luo J, Wang T, Ren J, Hu K, Wu G (2012). The activation of protease-activated receptor 1 mediates proliferation and invasion of nasopharyngeal carcinoma cells. Oncol Rep.

[108] Fazzini A, D'Antongiovanni V, Giusti L, Da Valle Y, Ciregia F, Piano I, Caputo A, D'Ursi AM, Gargini C, Lucacchini A, Mazzoni MR (2014). Altered protease-activated receptor-1 expression and signaling in a malignant pleural mesothelioma cell line, NCI-H28, with homozygous deletion of the beta-catenin gene. PLoS One.

[109] Goyama S, Shrestha M, Schibler J, Rosenfeldt L, Miller W, O'Brien E, Mizukawa B, Kitamura T, Palumbo JS, Mulloy JC (2017). Protease-activated receptor-1 inhibits proliferation but enhances leukemia stem cell activity in acute myeloid leukemia. Oncogene.

[110] Li SM, Jiang P, Xiang Y, Wang WW, Zhu YC, Feng WY, Li SD, Yu GY (2014). Protease-activated receptor (PAR)1, PAR2 and PAR4 expressions in esophageal squamous cell carcinoma. Dongwuxue Yanjiu.

[111] Kuhn SA, Martin M, Brodhun M, Kratzsch T, Hanisch UK, Haberl H (2014). Overexpression of protease-activated receptor type 1 (PAR-1) in glioblastoma multiforme WHO IV cells and blood vessels revealed by NCAM-assisted glioblastoma border labeling. Neurol Res.

[112] Krenzlin H, Lorenz V, Alessandri B (2017). The involvement of thrombin in the pathogenesis of glioblastoma. J Neurosci Res.

[113] Xie Q, Bao X, Chen ZH, Xu Y, Keep RF, Muraszko KM, Xi G, Hua Y (2016). Role of Protease-Activated Receptor-1 in Glioma Growth. Acta Neurochir Suppl.

[114] Auvergne R, Wu C, Connell A, Au S, Cornwell A, Osipovitch M, Benraiss A, Dangelmajer S, Guerrero-Cazares H, Quinones-Hinojosa A, Goldman SA (2016). PAR1 inhibition suppresses the self-renewal and growth of A2B5-defined glioma progenitor cells and their derived gliomas in vivo. Oncogene.

[115] Mussbach F, Henklein P, Westermann M, Settmacher U, Bohmer FD, Kaufmann R (2015). Proteinase-activated receptor 1- and 4-promoted migration of Hep3B hepatocellular carcinoma cells depends on ROS formation and RTK transactivation. J Cancer Res Clin Oncol.

[116] Martin F, Long JC, O'Toole SA, O'Leary JJ, Abu Saadeh F, Gleeson N, Norris LA (2016). PO-14 - Tumour expression of coagulation proteases of the aPC pathway - a role in the pathogenesis of gynaecological cancers?. Thromb Res.

