# Supplementary material for: Protease-activated receptor-1 (PAR-1): a promising molecular target for cancer
Source: Oncotarget. 2017 Sep 18;8(63):107334–45. doi: 10.18632/oncotarget.21015 (PMC5739818; doi:10.18632/oncotarget.21015)
Supplement: Supplementary file 1 [file oncotarget-08-107334-s001.pdf]

Protease-activated receptor-1 (PAR-1): a promising molecular target for cancer

SUPPLEMENTARY MATERIALS

Supplementary Table 1: PAR-1 in cancer. See Supplementarty_Table_1
