# Supplementary material for: Protease-activated receptor-1 (PAR-1): a promising molecular target for cancer
Source: Oncotarget. 2017 Sep 18;8(63):107334–45. doi: 10.18632/oncotarget.21015 (PMC5739818; doi:10.18632/oncotarget.21015)
Supplement: Supplementary file 2 [file oncotarget-08-107334-s002.docx]

**Supplementary Table 1: PAR-1 in cancer**

| **Cancer** | **PAR-1 function role and cellular effect** | **Referrence** |
| --- | --- | --- |
| Breast cancer | Highly expressed in invasive breast carcinoma cell lines | 38,70,71 |
|  | Activated by thrombin，MMP and TF, act with PAR-2 | 8,72 |
|  | Mediated breast cancer progression and invasion, essential for tumor growth and invasion with EGFR and ErbB/Her2 or by the fibroblast-derived MMP-1 mediated Ca^2+^ pathway | 8,36 |
| Melanoma | Highly expressed both in metastastatic melanoma cell lines and in metastatic lesions | 11,55 |
|  | Melanoma cells isolated from patients’ metastatic lesions had increased PAR-1 mRNA and protein expression compared to those of non-metastatic disease | 73 |
|  | PAR-1 signaling mobilized adhesion, invasion, anti-apoptotic and angiogenic factors to promote the invasion and metastasis of melanoma | 11,32,40,73 |
|  | The migration capability of melanoma cells is enabled by thrombin- or MMP-1-mediated PAR-1 activation | 40,70,74,75 |
|  | MMP-1 is shown to enhance type I collagen levels through skin to promote melanoma invasion, whereas PAR-1 activation leads to an increase in growth factor activation of EGFR and IGF-1 | 40,55 |
|  | PAR-1 induces metastatic melanoma by modulating tumor suppressor Maspin and the connexin 43 | 76 |
|  | PAR-1 silencing and inhibiting thrombin decrease dissemination of metastatic melanoma cells | 11,33,77 |
|  | PAR-1 siRNA mediated inhibition decreased MMP-2, IL-8 and VEGF, expression levels, subsequently vascular density | 78 |
| Renal cell cancer | PAR-1 was associated with distant metastasis and survival. AA genotype of PAR-1 gene variant IVSn-14A> T was associated with an increased risk of Renal cell cancer metastasis and a poorer prognosis | 79 |
|  | STAT3-dependent EGFR and PAR-1 activation in endothelial cells OF clear cell renal cell carcinoma was significantly increased | 55 |
| Gastric | Thrombin-induced PAR-1 activation EMT | 80 |
| cancer | Over expression of NF-kB,EGFR and TN-C activate PAR-1 expression, which could promote tumor cell growth and invasion | 54 |
|  | PAR-1 deeply participates in the ability of multi drug resistance and tumorigenesis through interaction with the Hippo-YAP pathway signaling | 43 |
|  | EPCR contributes to the proliferation and migration of MGC803 gastric cancer cells by activating ERK^1/2^, and this effect of EPCR may be dependent on PAR-1 | 37 |
|  | PAR-1 and MMP-1 up-regulating Galectin-3 in gastric cancer metastasis | 41 |
|  | ncRuPAR regulate PAR-1 and VEGF in GC patients | 59 |
|  | ALEX1, a novel tumor suppressor gene,inhibits gastric cancer metastasis via the PAR-1/Rho GTPase signaling pathway | 81 |
|  | PAR-1 expression levels are higher in metastatic gastric cancer and have prognostic value | 82 |
| Colorectal cancer | PAR-1 associated with prognostic factors for colorectal cancer, could promote colorectal cancer growth, local invasion and metastasis | 83,84 |
|  | Downregulation of a long noncoding RNA-ncRuPAR contributes to tumor inhibition through PAR-1 and VEGF in colorectal cancer | 85 |
|  | Stimulated platelet activation in the EMT and migration | 86 |
|  | Thrombin-induced HIF-1α increased Twist mRNA and its protein level was mediated by the modulation of PAR-1 activation and the HIF-1α translational pathway, which can regulate epithelial-mesenchymal transition (EMT) and increase tumor metastasis | 42 |
|  | KLK4 induced PAR-1 signaling in colon tumorigenesis | 87 |
|  | Tumor-endothelial cross-talk via an intravascular MMP1/PAR1 axis in microvascular and macrovascular endothelium | 88 |
|  | Commutators of PAR-1 signaling in cancer cell invasion reveal an essential role of the Rho-Rho kinase axis and tumor microenvironment | 89 |
|  | Activation of PAR-1 promotes human colon cancer cell proliferation through EGFR transactivation | 90 |
| Lung cancer | PAR-1 have a diagnostic value ,it is expressed on cells that constitute the pulmonary tumor microenvironment, including vascular endothelial cells, macrophages and stromal fibroblasts, is associated with advanced tumor stages and, in with shorter median OS in squamous cell lung carcinoma | 91,92,93 |
|  | Cancer-associated VTE and cancermortality ,through activate PAR-1 by whole blood flow cytometry | 94 |
|  | TGFβ upregulates PAR-1 expression and signalling responses in A549 lung adenocarcinoma cells | 96 |
|  | Gαq and Gα₁₃ coupled PAR-1 and constitutively active GαqQL and Gα₁₂/₁₃QL mutants stimulated Gli in SCLC linking autocrine BBS and Shh circuitries | 97 |
| Pancreatic cancer | PAR-1 expression is associated with disease progression and overall survival in pancreatic cancer | 98,99,100 |
|  | Thrombin significantly enhanced adhesion of pancreatic cancer cells to vitronectin through PAR- 1 depending on the presence of integrin β1. | 101 |
|  | Nuclear Ca ^2+^ signaling generated by trypsin and thrombin promote PAR-1 to cell proliferation | 39 |
| Prostate cancer | PAR-1 is overexpressed in prostate cancer, may contribute to the malignant progression of prostate cancer | 102,103 |
|  | MMP-1 and PAR-1 coexpression with the clinicopathological characteristics and prognosis | 103 |
|  | TK promotes keratinocyte migration through activation of PAR-1 and transactivation of EGFR | 104 |
|  | Cancer epithelium produces KLK4 to activate PAR-1 in the surrounding stroma, which in-turn releases cytokines (IL-6) that stimulate cancer cells to proliferate and increase production of KLKs | 105 |
| Nasopharyngel cacinoma | PAR-1 may contribute to the growth and invasive potential of Nasopharyngeal cacinoma | 106,107 |
|  | Thrombin-induced PAR-1 activation breaks down extracellular matrix and basement membrane to increase MMP-1/-9 levels | 107 |
| Acute myeloid leukemia | [PAR-1 inhibits proliferation but enhances leukemia stem cell activity in acute myeloid leukemia.](https://www.ncbi.nlm.nih.gov/pubmed/27819671) | 108,109 |
| Esophageal squamous cell carcinoma | The expression of PAR-1 in esophageal squamous cell carcinoma was increased | 110,111 |
| Glioblastoma | Thrombin activates PAR-1 expression, thus enabling tumor cell seeding and metastasis, giving rise to increased tumor cell growth and angiogenesis | 112 |
| Gliomas | Per HIF-α/VEGF pathway, PAR-1 maintains self-renewal and tumorigenicity of tumor-initiating progenitor cells (TPC) in gliomas, whilst inhibition of PAR-1 signaling slows down tumor progression | 113,114 |
| Hepatocellular carcinoma | PAR-1 and PAR-4 activate common promigratory signaling pathways in Hep3B liver carcinoma cells | 115 |
| Ovarian cancer | PAR-1 is associated with the pathogenesis of ovarian cancer | 116 |
|  | associated with PO-14 - tumor expression of coagulation proteases of the APC pathway | 116 |
